# Supplementary material for: A G1‐like state allows HIV‐1 to bypass SAMHD1 restriction in macrophages
Source: EMBO J. 2017 Jan 25;36(5):604–16. doi: 10.15252/embj.201696025 (PMC5331754; doi:10.15252/embj.201696025)
Supplement: Supplementary file 1 — Expanded View Figures PDF [file EMBJ-36-604-s001.pdf]

Expanded View Figures

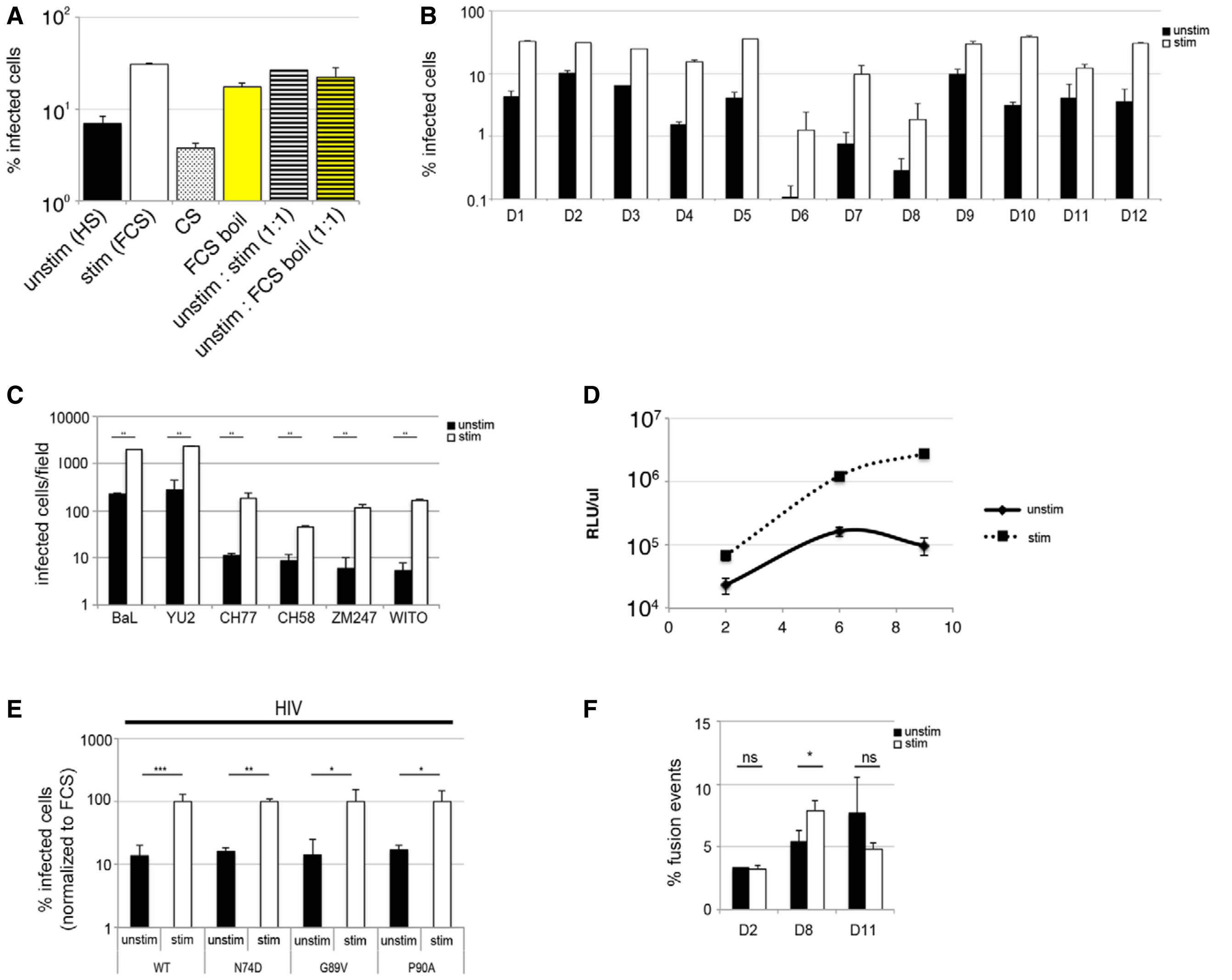

Figure EV1. Enhanced susceptibility to HIV-1 infection in stimulated monocyte-derived macrophages.

A MDM were differentiated in RPMI complemented with MCSF and 10% human serum for 3 days and cultured for additional 3 days in RPMI complemented with 10% human serum (unstim), 10% foetal calf serum (stim), 10% charcoal-stripped FCS (CS), 10% FCS boiled for 5 min and filtrated through 0.45-μm filter (FCS boil), 1:1 mix of 10% HS and FCS (unstim:stim 1:1), 1:1 mix of 10% HS and FCS boiled for 5 min (unstim:FCS boil 1:1) and then infected with VSV-G-pseudotyped HIV-1-expressing GFP. The percentage of infected cells was detected 48 h post-infection by FACS.

B MDM from 12 different donors (D1-D12) were infected with VSV-G-pseudotyped HIV-1 GFP; the percentage of infected cells was detected 48 h post-infection by FACS.

C Single round of infection of MDM with a panel of full-length HIV-1 viruses (macrophage tropic viruses: BaL and YU2; clinical isolates: CH77, CH58, ZM247, WITO). Cells were stained for intracellular p24 protein 48 h post-infection and numbers of infected cells quantified by light microscopy.

D The titres of released viruses were determined by infection of the indicator cells HeLa TZM-bl. Culture supernatants from BaL-infected MDM were harvest at day 2, 6 and 9 post-infection, filtered and used to infect TZM-bl cells. Luciferase activity of the TZM-bl cells was measured 24 h post-infection.

E MDM were infected with VSV-G-pseudotyped GFP viruses (wt HIV-1; HIV-1 capsid mutants P90A, N74D, G89V), and the percentage of infected cells was detected by FACS 48 h post-infection. All viruses displayed similar percentage of infection (which was normalised to infection in stimulated macrophages ~100%).

F MDM were infected with equal amounts of p24 (50 ng) of BlaM-Vpr-containing viruses for 4 h. Cells were loaded with CCF2/AM dye, and fusion events were detected by flow cytometry using BD LSR Fortessa and gated from 10,000 cells.

Data information: Graphs are average of $n \geq 3$, mean ± s.e.m.; *P-value ≤ 0.05; **P-value ≤ 0.01; ***P-value ≤ 0.001, unpaired *t*-test.

 © 2017 The Authors

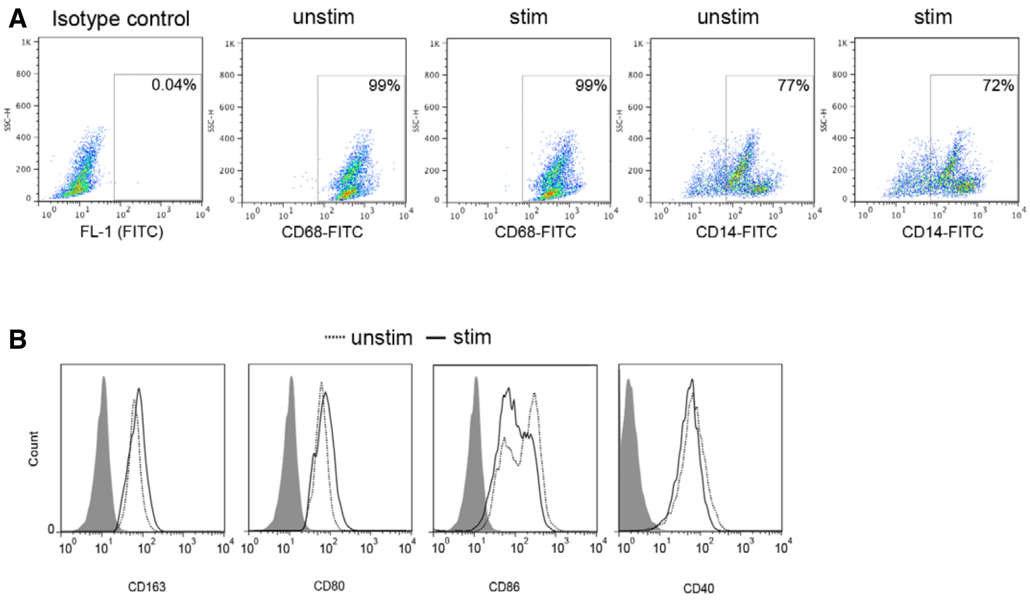

Figure EV2. Expression of macrophage cell surface markers is unaffected by stimulatory conditions.

A, B Stimulated or unstimulated MDM were stained for classical macrophage markers (CD68, CD14) and M1 and M2 macrophage markers (CD163, CD80, CD86 and CD40) and analysed by FACS.

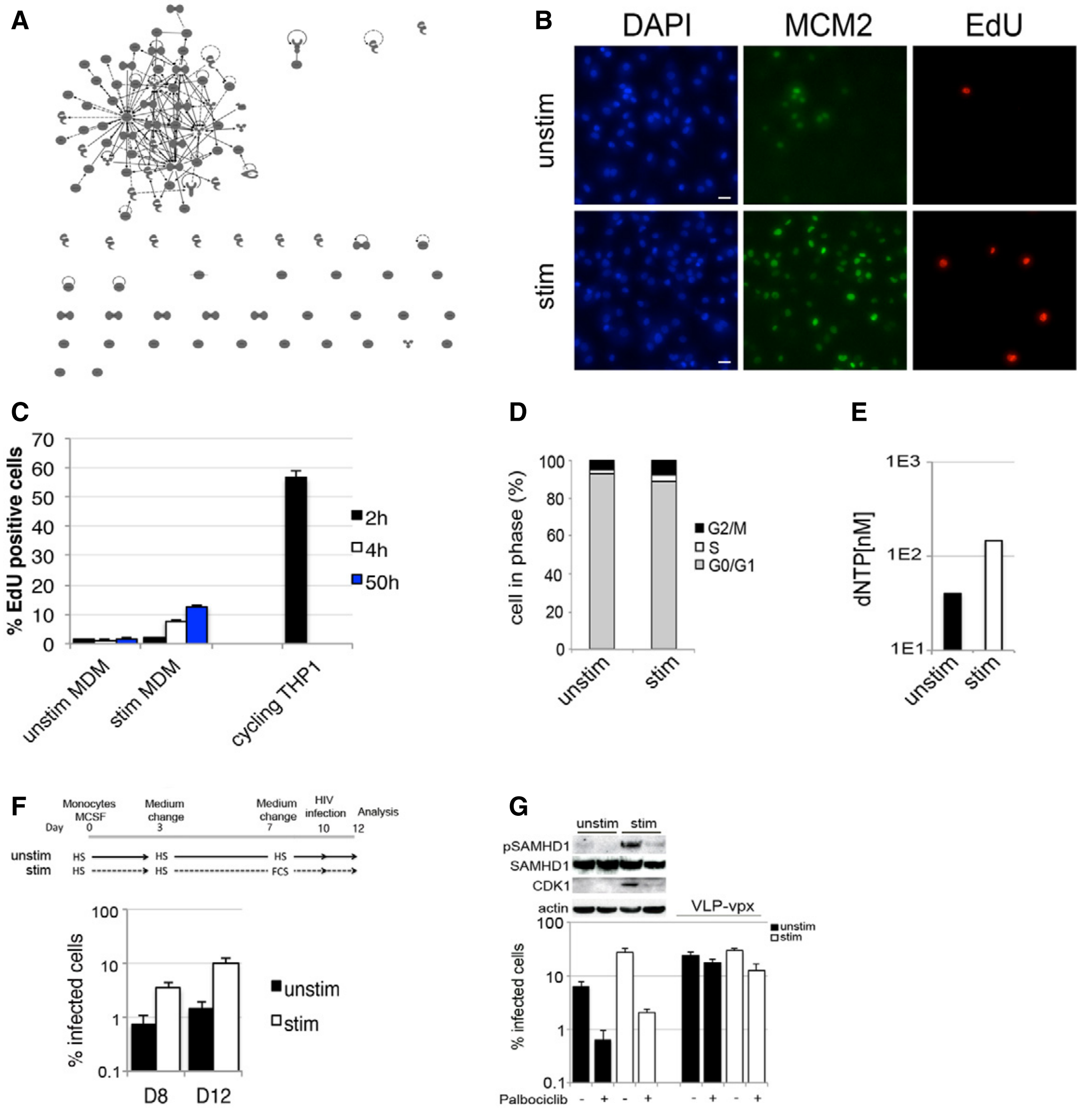

Figure EV3. MDM can be manipulated to transition between G0- and a G1-like phase.

A Ingenuity pathway interaction analysis of nuclear proteins that show significant transcriptional upregulation in stimulated MDM compared to unstimulated MDM. The figure demonstrates a single dominant cluster of interacting proteins.

B Example of acquisition by Hermes WiScan cell-imaging system, an automated microscopic platform. MDM were labelled for nuclei, MCM2 and active DNA synthesis using Click-iT® EdU Alexa Fluor® 488 Imaging Kit. Scale bars: 10 μm.

C MDM were treated for 2, 4 and 50 h with 10 μM EdU to detect active DNA synthesis using Click-iT® EdU Alexa Fluor® 488 Imaging Kit. The percentage of EdU-positive cells was quantified by FACS.

D Quantification of cell cycle phases from PI labelling.

E dNTP levels in stimulated and unstimulated MDM.

F MDM differentiated and cultured in RPMI complemented with MCSF and 10% human serum for 7 days were changed into stimulatory medium (10% FCS) and cultured for additional 3 days. Cells were infected with VSV-G-pseudotyped HIV-1 GFP and the percentage of infected cells detected 48 h post-infection by FACS.

G MDM were treated with the CDK4/6 inhibitor Palbociclib (1 μM) 18 h before infection and infected with VSV-G-pseudotyped GFP virus; VLP-vpx was added at the time of infection. Cells from this experiment were lysed and used for immunoblotting.

Data information: Cells in each experiment were recorded and analysed using Hermes WiScan cell-imaging system, ImageJ or FACS ($n \geq 3$, mean ± s.e.m.).

© 2017 The Authors

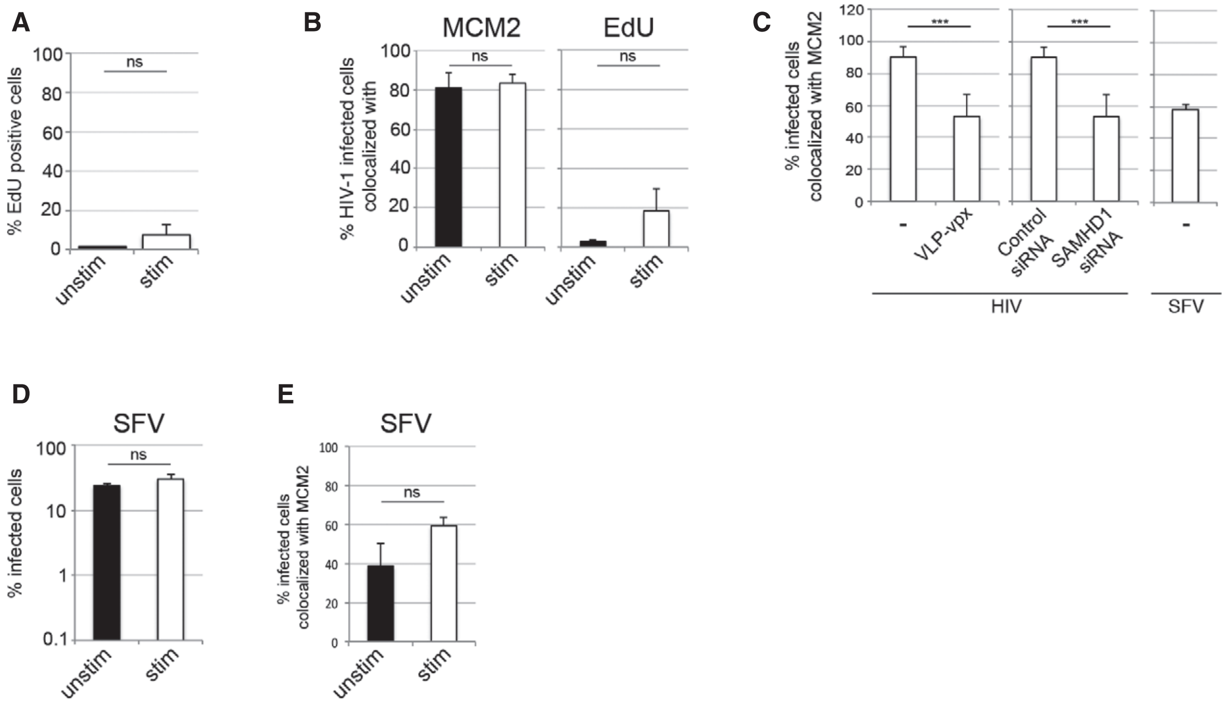

Figure EV4. MDM in a G1-like phase are highly permissive to HIV-1 infection.

A MDM were cultured in the presence of 5 μM EdU for 72 h and labelled using Click-iT® EdU Alexa Fluor® 488 Imaging Kit. The percentage of EdU-positive cells was detected by using Hermes WiScan and ImageJ.

B, C MDM were infected with VSV-G-pseudotyped HIV-1 GFP or Semliki Forest virus (SFV) stained and recorded 48 h post-infection and analysed for co-localisation between infection and MCM2 protein or EdU incorporation (active DNA synthesis, EdU was added to cells at the time of infection). Co-localisation analysis between infection and MCM2 in the presence or absence of SAMHD1 protein is also shown.

D MDM infection by SFV.

E Co-localisation between SFV infection and MCM2 protein.

Data information: On average, 10^4 cells in each experiment were recorded and analysed using Hermes WiScan cell-imaging system and ImageJ ($n \geq 3$, mean ± s.e.m.; ***P-value ≤ 0.001, unpaired t-test).

© 2017 The Authors

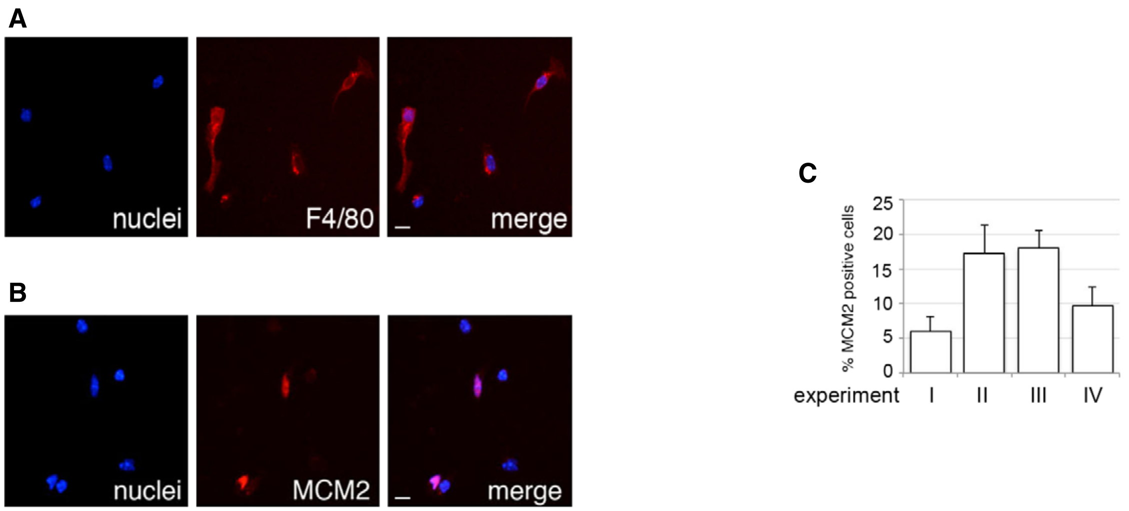

Figure EV5. Microglia can be observed in a G1-like phase.

A, B Microglia isolated from 8- to 12-week-old CD1 mice were stained for the macrophage markers (A) F4/80 and (B) MCM2 4 h after isolation. Scale bars: 10 μm.

C Graph shows the percentage of MCM2-positive cells from four independent experiments. On average, 2×10^3 cells in each experiment were recorded and analysed using Hermes WiScan cell-imaging system and ImageJ (mean ± s.e.m.).

 © 2017 The Authors

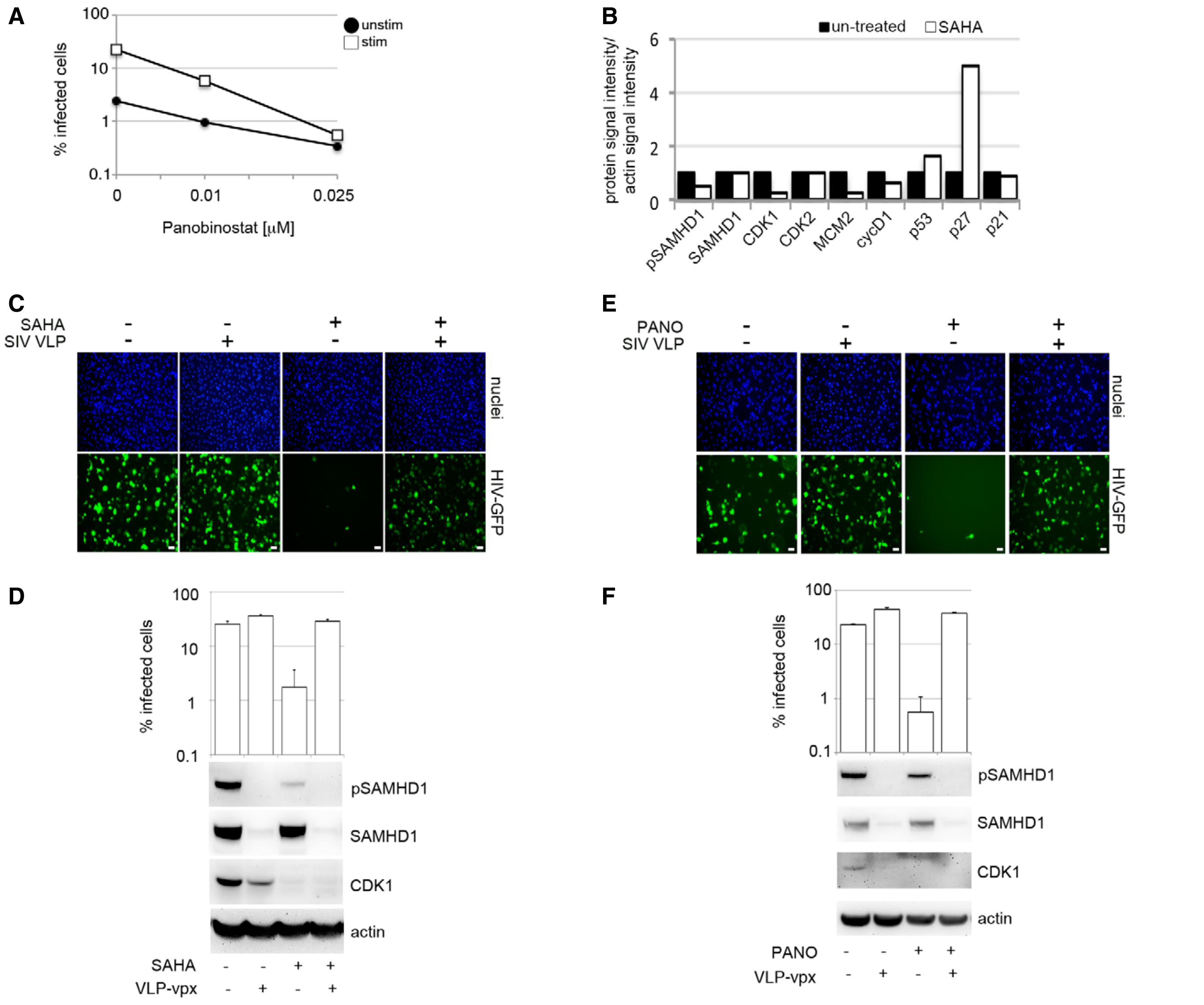

Figure EV6. HDAC inhibitor blockade of HIV-1 infection is SAMHD1-dependent.

A MDM were treated with panobinostat (PANO) 18 h before infection with VSV-G-pseudotyped HIV-1 GFP. The percentage of infected cells was detected by FACS 48 h post-infection.

B Quantification of specific protein band intensities from the immunoblot in Fig 5D using a CCD camera. Intensities of proteins bands were normalised to intensity of the actin protein band.

C MDM were treated with 1 μM SAHA (Vorinostat), and then recorded and analysed for infection (VSV-G HIV-GFP) using Hermes WiScan. Scale bars: 20 μm.

D MDM were treated with 1 μM SAHA (Vorinostat) and co-infected with VSV-G-pseudotyped HIV-1 GFP and VLP-vpx. Cells from a representative donor were lysed and used for immunoblotting. The percentage of infected cells was detected by Hermes WiScan.

E MDM were treated with 0.025 μM panobinostat and then recorded and analysed for infection (VSV-G HIV-GFP) using Hermes WiScan. Scale bars: 20 μm.

F MDM were treated with 0.025 μM panobinostat and co-infected with VSV-G-pseudotyped HIV-1 GFP and VLP-vpx. Cells from a representative donor were lysed and used for immunoblotting. The percentage of infected cells was detected by Hermes WiScan.

Data information: On average, 10^4 cells in each experiment were recorded and analysed using Hermes WiScan cell-imaging system and ImageJ ($n \geq 3$, mean ± s.e.m.). Source data are available online for this figure.

© 2017 The Authors
