# Supplementary material for: A G1‐like state allows HIV‐1 to bypass SAMHD1 restriction in macrophages
Source: EMBO J. 2017 Jan 25;36(5):604–16. doi: 10.15252/embj.201696025 (PMC5331754; doi:10.15252/embj.201696025)
Supplement: Supplementary file 2 — Table EV1 [file EMBJ-36-604-s002.zip › EMBOJ_96025_TableEV1/EJ_96025_TableEV1_legend.docx]

**Table EV1 - Transcriptomic analysis of stimulated and unstimulated MDM.**

mRNA with >two‑fold mean expression differences between stimulated and unstimulated MDM are shown.
