# Supplementary figures and images for: A G1‐like state allows HIV‐1 to bypass SAMHD1 restriction in macrophages
Source: EMBO J. 2017 Jan 25;36(5):604–16. doi: 10.15252/embj.201696025 (PMC5331754; doi:10.15252/embj.201696025)

Fig EV6D

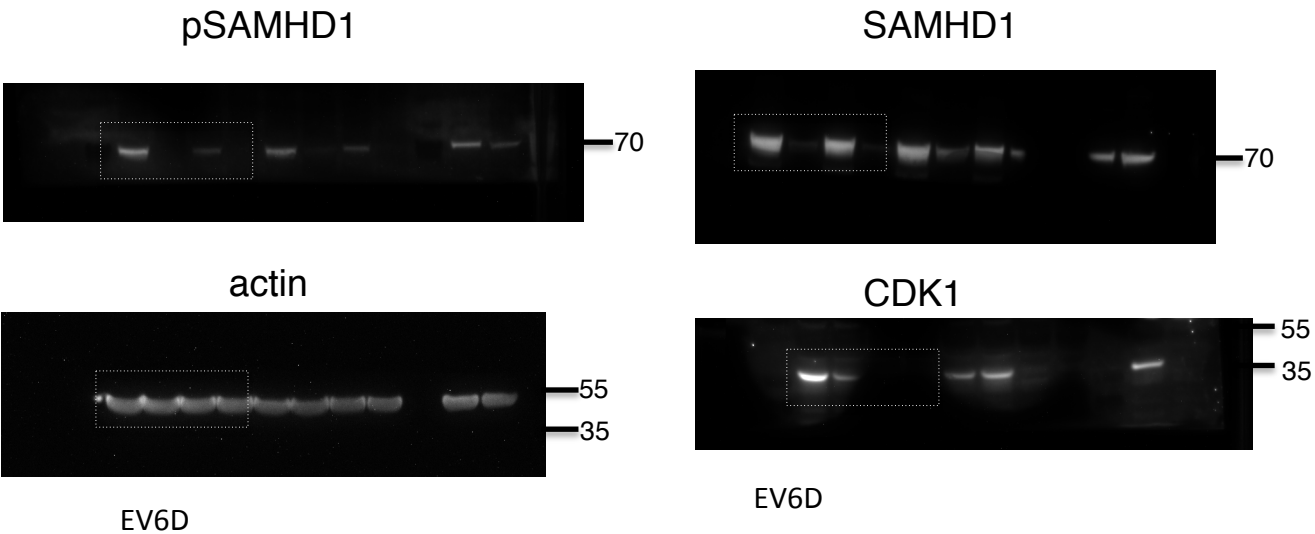

Fig EV6F

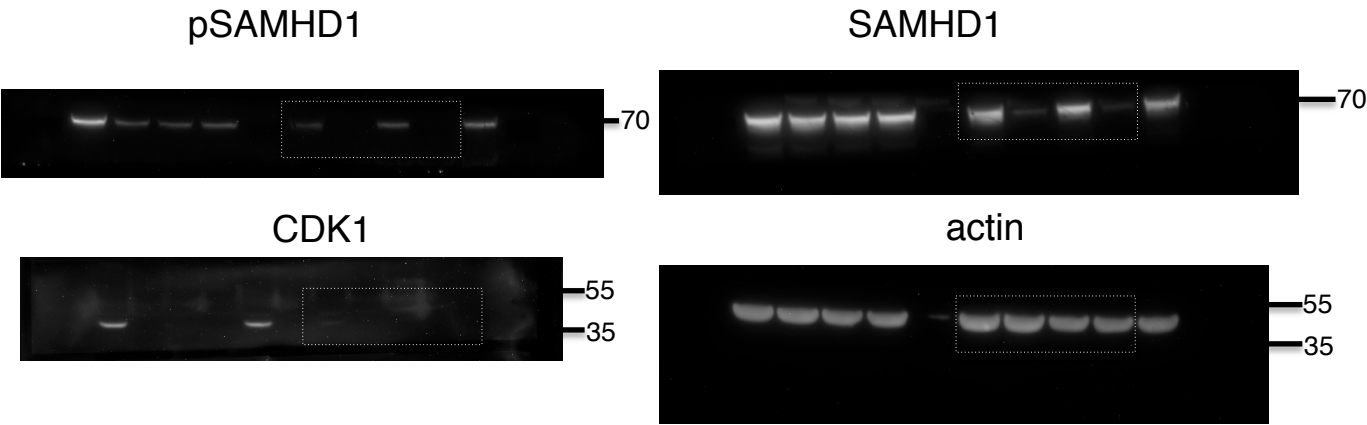

Supplement: Supplementary file 3 — Source Data for Expanded View [file EMBJ-36-604-s003.zip › source_data_FIG_EV6.pdf]

Fig 1G

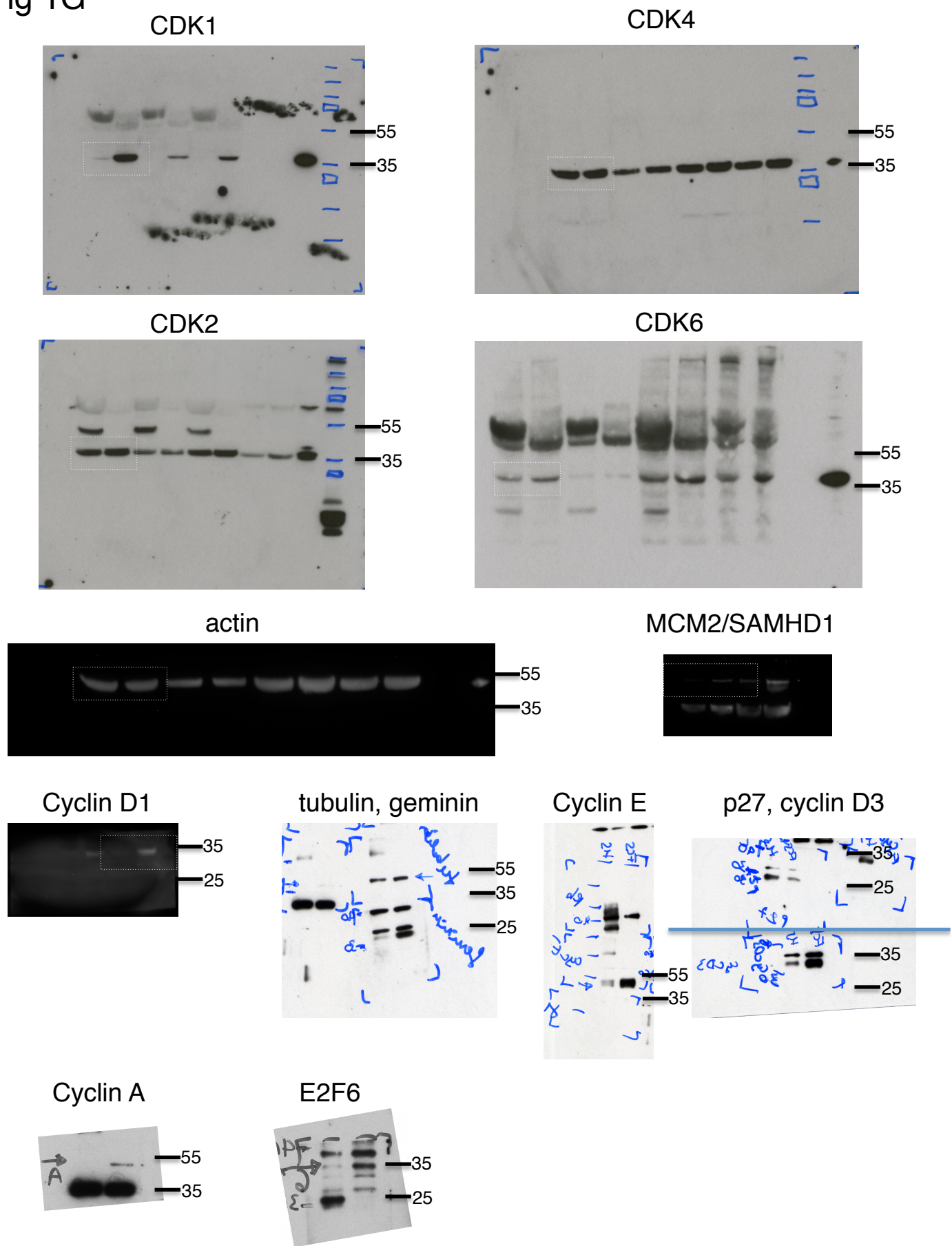

Supplement: Supplementary file 5 — Source Data for Figure 1 [file EMBJ-36-604-s004.pdf]

Fig 2 A

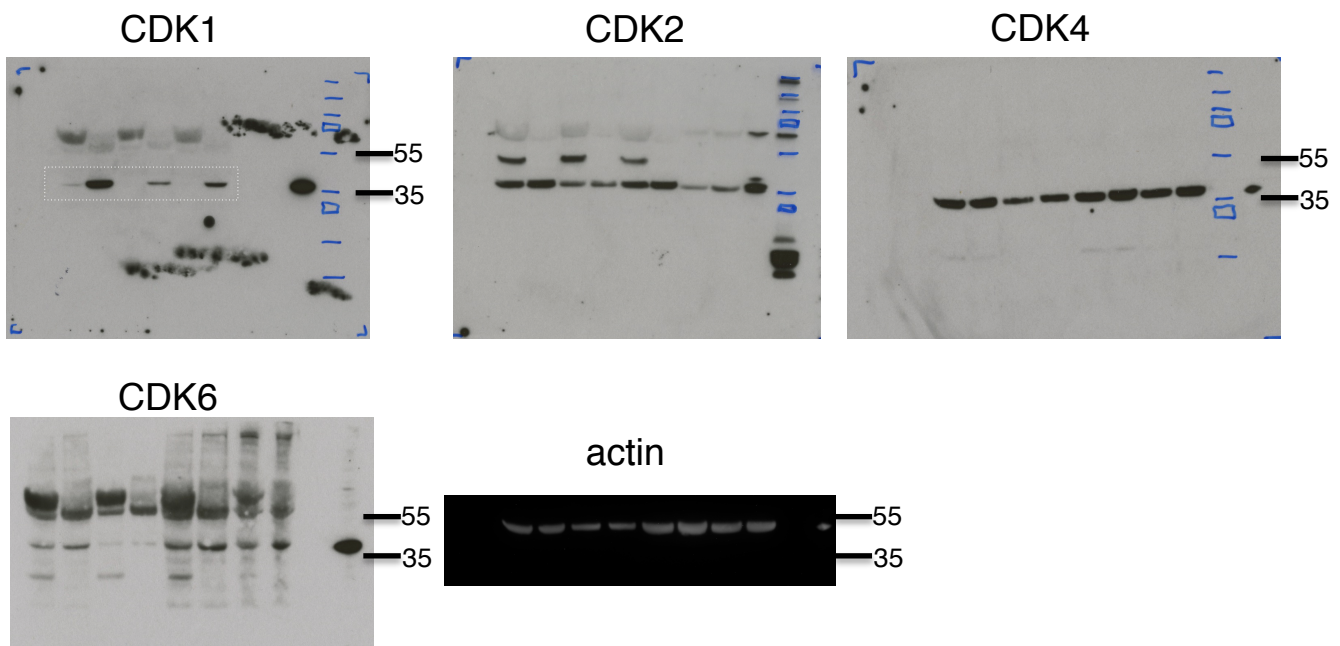

Fig 2 B

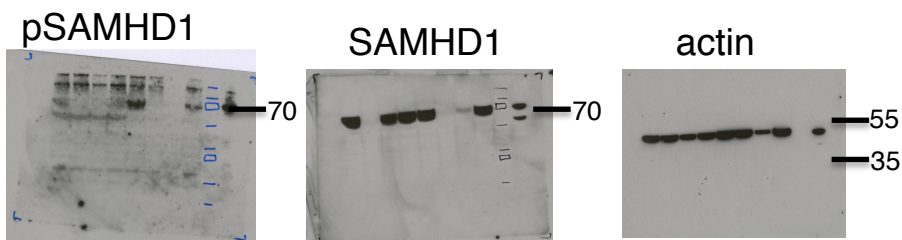

Fig 2C

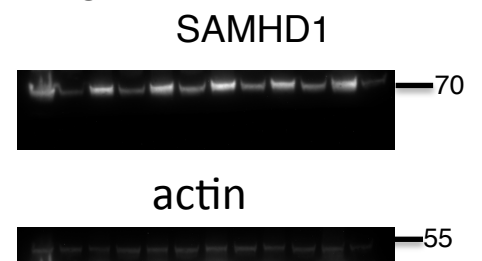

Fig 2 E and I

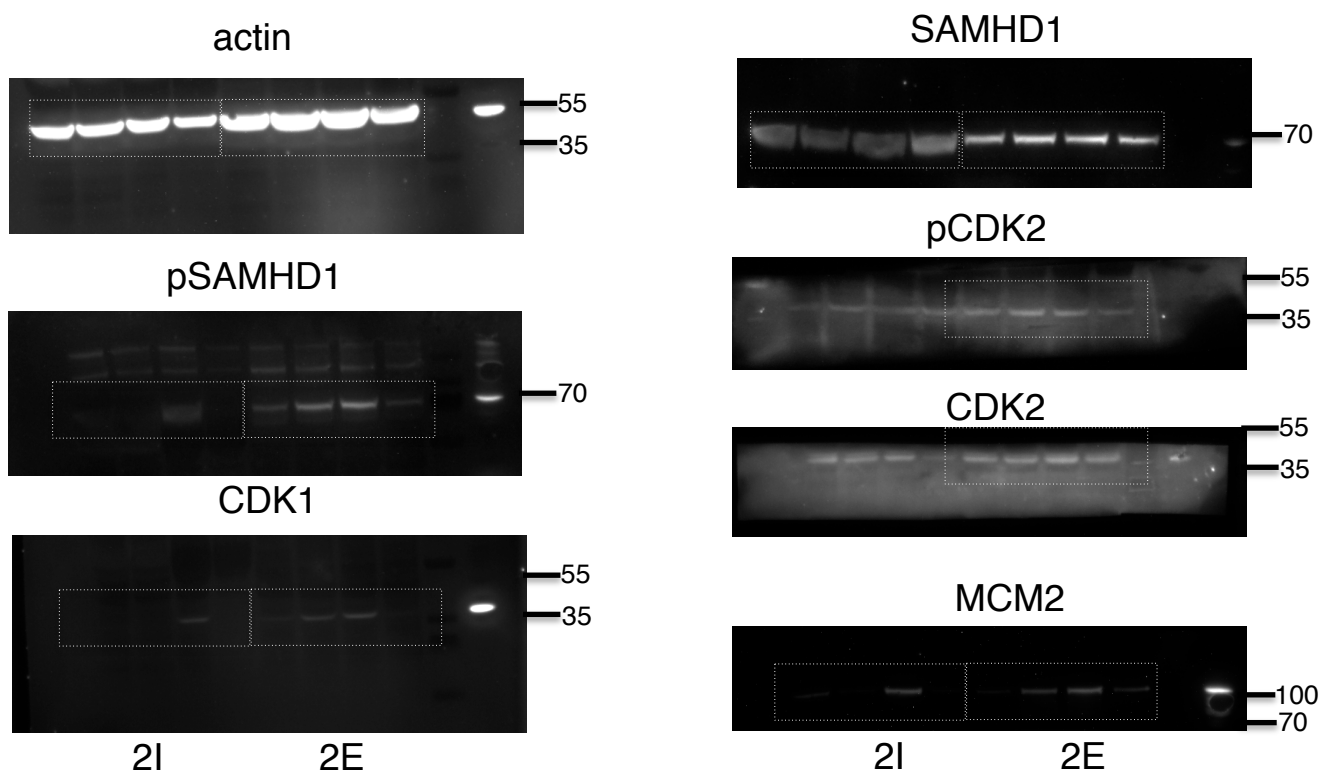

Supplement: Supplementary file 6 — Source Data for Figure 2 [file EMBJ-36-604-s005.pdf]

Fig 5D

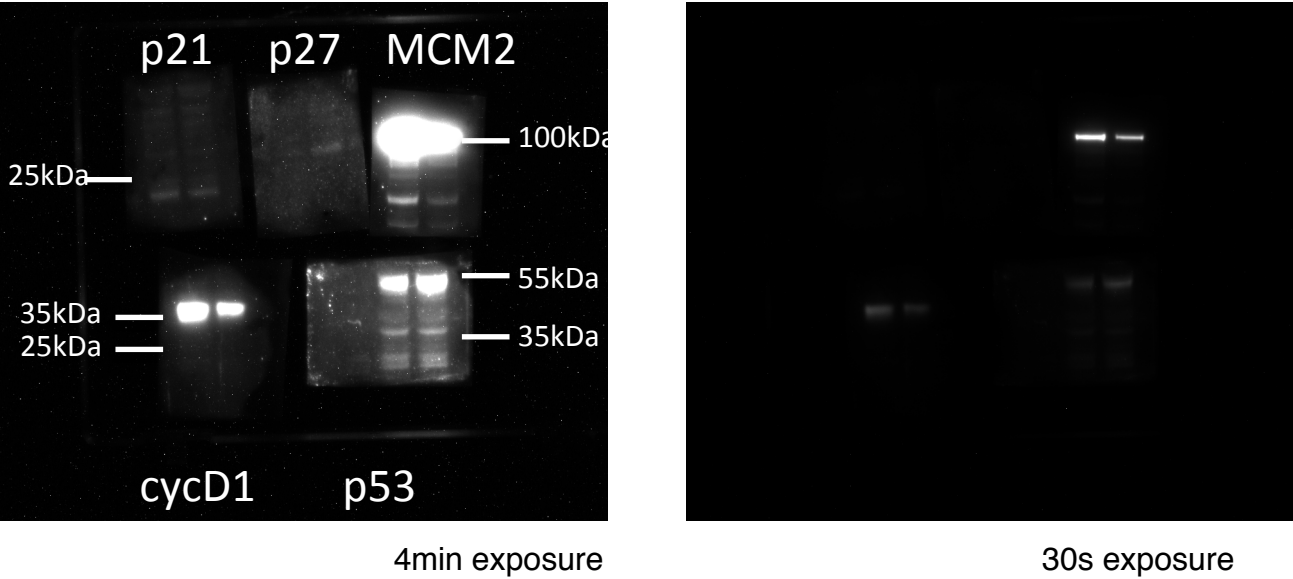

Fig 5E

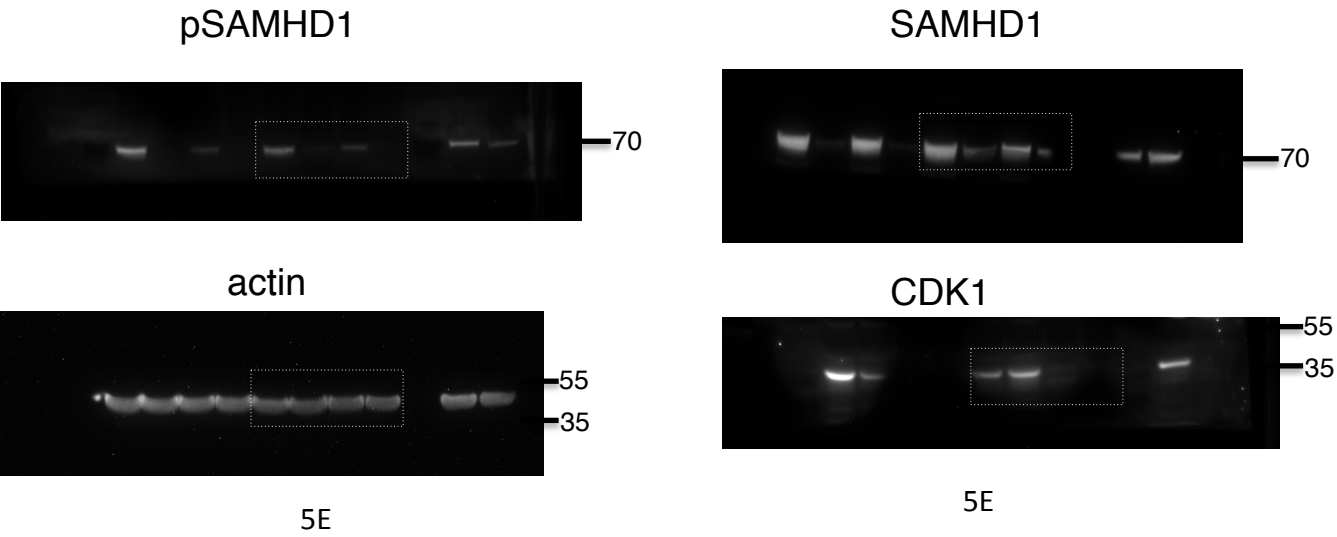

Supplement: Supplementary file 7 — Source Data for Figure 5 [file EMBJ-36-604-s006.pdf]
